# Impact of grain orientation and phase on Volta potential differences in an additively manufactured titanium alloy

**DOI:** 10.1063/5.0038114

**Published:** 2021

**Authors:** Jake T. Benzing, Olivia O. Maryon, Nik Hrabe, Paul H. Davis, Michael F. Hurley, Frank W. DelRio

**Affiliations:** 1 Material Measurement Laboratory, National Institute of Standards and Technology, Boulder, Colorado 80305, USA; 2 Micron School of Materials Science and Engineering, Boise State University, Boise, Idaho 83725, USA

## Abstract

This work introduces a method for co-localized multi-modal imaging of sub-*μ*m features in an additively manufactured (AM) titanium alloy. Ti-6Al-4V parts manufactured by electron beam melting powder bed fusion were subjected to hot isostatic pressing to seal internal porosity and machined to remove contour–hatch interfaces. Electron microscopy and atomic force microscopy-based techniques (electron backscatter diffraction and scanning Kelvin probe force microscopy) were used to measure and categorize the effects of crystallographic texture, misorientation, and phase content on the relative differences in the Volta potential of *α*-Ti and *β*-Ti phases. Given the tunability of additive manufacturing processes, recommendations for texture and phase control are discussed. In particular, our findings indicate that the potential for micro-galvanic corrosion initiation can be regulated in AM Ti-6Al-4V parts by minimizing both the total area of {111} prior-β grains and the number of contact points between {111} β grains and *α* laths that originate from {001} prior-β grains.

Titanium alloys are prized in aerospace applications due to their combination of high strength-to-weight ratio and excellent corrosion resistance.^1-3^ Additionally, titanium’s excellent biocompatibility enables the use of titanium alloys in biomedical applications.^4,5^ Ti-6Al-4V is the most commonly manufactured titanium alloy and is ranked with high importance in the additive manufacturing (AM) community.^6^ Recently, AM has been employed to fabricate complex shapes for dental applications and other medical implants^7-15^ by melting Ti-6Al-4V powder in a layer-by-layer fashion. Although AM parts typically contain pores and other defects,^11^ recent work has shown that Ti-6Al-4V parts produced via electron beam melting powder-bed fusion (EBM-PBF) can replace wrought components if porosity is removed by hot isostatic pressing (HIP)^16,17^ and phase content/grain size is optimized.^18-20^ An advantage of AM is that microstructures and properties can be tuned locally during the manufacturing process.^21^ While previous studies show how the corrosion resistance of titanium alloys can be manipulated with alloying,^22-24^ this can be difficult to implement in AM since only a few powder compositions have been optimized for manufacturing.

The corrosion behavior of AM Ti-6Al-4V has previously been analyzed on a bulk scale (e.g., potentiodynamic polarization tests and standardized methods for measuring open circuit potentials between electrodes) to determine the effects of as-built surfaces, heat treatment temperature, corrosive environment temperature, and corrosive solution concentration.^7,16,18,19^ While grain size was a microstructural consideration, crystallographic texture was not scrutinized in previous work. The latter can convolute discussion since there is evidence of large texture variation in EBM-PBF Ti-6Al-4V.^25^ In addition, a previous study^26^ on EBM-PBF Ti-6Al-4V showed that a sub *β*-transus HIP treatment increased the fraction of *β*-phase but caused little to no variation in the relative peak intensity of *α*-phase poles measured with x-ray diffraction. Therefore, it would be advantageous to control corrosion behavior by manipulating tunable microstructural features of interest, such as texture or phase fraction, as opposed to changing powder chemistry. Recently, scanning Kelvin probe force microscopy (SKPFM) has been used to investigate the local influences of chemistry and phase content in Ti-6Al-4V.^27^ This high spatial resolution, nondestructive imaging technique can generate nanoscale maps of Volta potential, which can be a predictor of corrosion initiation.^27-34^ When relating Volta potential measurements to anodic and cathodic behavior, the separation distance has two known effects, depending upon the length scale of interest. First, since *α*-Ti and *β*-Ti have an ≈15% difference in the atomic percent of vanadium,^35^ micro-scale galvanic corrosion may be the main driving force between the two phases present within Ti-6Al-4V. Second, macro-scale couplings may come into play if microstructural features such as crystallographic texture are varied at a large length scale or if the part contacts a different material/environment.

In this work, Ti-6Al-4V parts manufactured via EBM-PBF were subjected to HIP treatment and subsequently characterized using multiple imaging techniques. Crystallographic information measured by electron backscatter diffraction (EBSD) was aligned with SKPFM maps of the same region to measure the influences of grain orientation, misorientation, and phase content on the Volta potential. While phase fraction produces a noticeable difference in Volta potential, an important new contribution from this work is the clear identification of significant differences in Volta potential based on grain orientations for both *α*-Ti and *β*-Ti phases (hexagonal close-packed and body-centered-cubic crystal structures). Given the tunability of AM technologies, contributions to the literature are manifested through recommendations for texture control and are discussed in the context of regulating the Volta potential in regions of interest across multiple length scales.

Ti-6Al-4V parts (35 mm tall, 25 mm wide, and 15 mm thick) were fabricated by EBM-PBF using an Arcam A1 machine (software version 3.2.132 kV, 60 kV accelerating voltage, 50 *μ*m layer thickness, speed factor 35) and Arcam Ti-6Al-4V gas-atomized powder (average diameter ≈70 *μ*m). The standard Ti-6Al-4V HIP cycle was used (2 h at 900 °C, 100 MPa in Ar, 12 °C/min heating and cooling rates) to seal internal porosity without considerably altering texture. Samples were analyzed for internal porosity with x-ray computed tomography (CT) (160 kV, 10 W, 1 *μ*m voxels). The samples contained the full spectrum of crystallographic texture variations previously observed.^25^

Samples were sectioned and polished using standard metal-lographic procedures (i.e., SiC paper, 1 *μ*m diamond particle suspension, and 50 nm colloidal silica). To co-localize measurements, an instrumented nano-indenter with a diamond Berkovich tip was used to create sets of three fiducial marks in the form of an asymmetric triangle. Secondary electron (SE) and backscattered electron (BSE) images were recorded from regions of interest using a field-emission scanning electron microscope (FE-SEM) at 20 keV and 8 mm working distance. EBSD measurements were performed in an FE-SEM at 20 keV and 19 mm working distance using 150 nm step size.

Topography and Volta potential maps were generated via SKPFM [Bruker Dimension Icon atomic force microscopy (AFM)] in a glovebox equipped with a gas purification unit. Inline sensors were used to monitor the inert argon atmosphere during measurements with levels of <0.1 ppm H_2_O and O_2_. The technique and procedure were described previously^29,36-38^ and is a proven method for minimizing surface contamination.^39^ A lift height of 25 nm was employed for the interleaved SKPFM pass, and the same Bruker PFQNE-AL probe whose Volta potential was calibrated with an Al/Si/Au standard was used throughout. Data were processed by applying a first-order flatten and second-order plane fit to all topography and Volta potential data (more details are provided in the supplementary material). When extracting additional measurements (line scans) from the EBSD and SKPFM maps, each interface (grain/phase boundary) was measured three times to give an average response. Within each 40 × 40 *μ*m^2^ field of view, approximately fifty unique interfaces were analyzed. An analysis of variance was completed with Instat software (Tukey test) and used to test the null hypothesis that Volta potentials were equal across grain orientations; significance was defined as p < 0.01.

Ti-6Al-4V blocks manufactured with EBM-PBF methods (Fig. 1) were subjected to a standard HIP treatment. No major differences in chemical composition were measured before and after HIP (Table I). The purpose of the standard HIP treatment was to remove internal porosity (confirmed with x-ray CT measurements in previous work^20,25^) as a confounding variable in the subsequent surface measurements, with the added benefit of a slight increase in grain size (compared to the as-built condition) for both *α* laths and *β* ribs/grains.

Figure 2 shows a representative region of interest (contains a wide variation in grain orientations) characterized via multiple techniques. Typical SEM images recorded with SE and BSE detectors are shown in Figs. 2(a) and 2(b), respectively, with the fiducial marks indicated and visible in the SE image [Fig. 2(a)]. Inverse pole figure (IPF) maps produced from EBSD measurements of the same region are separated by phase (*α*-Ti and *β*-Ti) in Figs. 2(c) and 2(d). Within this region, multiple SKPFM measurements were recorded to measure differences in height and Volta potential. A large overview SKPFM image is shown in Figs. 2(e) and 2(f). There are clear variations in Volta potential throughout the field of view that align with changes in grain orientation [Figs. 2(c) and 2(f)]. While this field of view was used, higher magnification SKPFM scans are provided in Figs. 3 and 4 to detail the methods used in characterizing *α* laths and *β* grains.

Figure 3 includes a BSE image next to a height map from SKPFM measurements. These were used to overlap the SEM-based and SKPFM-based datasets, with help from the fiducial mark. Although an SE image is more akin to the topographic information from an SKPFM height map, the BSE image provides better *α*–*β* contrast (due to differences in vanadium content). Most height differences are found on *α*–*β* boundaries and equate to a magnitude of ≈7 nm. The IPF map in Fig. 3(c) is shown with the corresponding SKPFM Volta potential map and indicates a clear relationship between grain orientation of *α* laths and Volta potential. A line scan across *α*–*α* grain boundaries indicates a misorientation of ≈60° and a relative Volta difference potential of 78 mV.

While the *α*–*β* differences are quantified in Fig. 4, it is important to note the orientation of *β* grains in the top-left portion of the images in Fig. 3(c), which are near {001}. The *β* grains are non-transformed portions of a larger prior-*β* grain, which is the first solid phase that forms after cooling from the liquid state. The *α* grains nucleate at lower temperatures, meaning that *α* orientations are governed by the *β* to *α* transformation. Thus, the orientation of *α* grains analyzed by the line scan in Figs. 3(c) and 3(e) is classified as *α* grains originating from a {001} prior-*β* grain.

A summary from all line scans [Fig. 3(g)] classifies *α* grains by prior-*β* grain orientation. The relative Volta potential differences [Fig. 3(g)] are grouped into three measurement categories: (i) within a single *α* lath, (ii) across *α*–*α* boundaries of similar grain orientation, and (iii) across *α*–*α* boundaries of differing grain orientation. Similar grain orientation is defined as when the average orientations of two neighboring grains (as plotted on an IPF triangle) are less than 5° away on an IPF plot. This use of degrees to separate or classify grain orientations as similar or different is not to be confused with the misorientation degrees that arise at grain boundaries, as defined later. Accordingly, the maximum and minimum Volta potential values measured in *α* grains arising from different prior-*β* grain orientations are shown in Fig. 3(h) and summarized in Table II. The most prominent trend is that Volta potential differences across *α*–*α* boundaries of differing grain orientation [category (iii)] are significantly greater than those from *α*–*α* boundaries of similar grain orientation [category (ii)]. In addition, *α* grains from {001} prior-*β* grains exhibit the lowest relative differences in Volta potential (Table II). While misorientations do not differ significantly within the same categories of *α* grain orientations, there are significant differences when comparing Volta potentials by prior-*β* grain orientation. When comparing the relative difference in potential within a single grain, across boundaries of differing orientations, and maximum potential, the {001} prior-*β* orientations are significantly different from the {101} and {111} prior-*β* orientations. In contrast, the orientation categories of {101} and {111} are not statistically different from each other for these *α*–*α* grain measurements and generally produce the largest relative differences in Volta potential as well as the greatest maximum Volta potential.

Figure 4 applies similar methods as Fig. 3 to *α*–*β* phase boundaries. While most of the bright regions in the BSE image [Fig. 4(a)] are likely *β* phase, care was taken to only make measurements across *β* grains that were confidently identified with EBSD at 150 nm step size. Based on the results in Figs. 4(g) and 4(h), which are summarized in Table III, *β* grains of near-{001} orientation exhibited the lowest maximum Volta potential (51 ± 10 mV), whereas *β* grains of near-{111} orientation exhibited a maximum Volta potential more than double (118 ± 12 mV) that of the {001} orientations (the differences are statistically significant). In fact, all combinations of maximum Volta potential differ significantly but with varying degrees of significance [p values in Fig. 4(h)]. The relative *α*–*β* Volta potential difference (difference between the average *α* Volta value and the average *β* Volta value) is the lowest for {001} *β* grains that neighbor *α* grains, which differ significantly from the other *β* grain orientation categories.

This work demonstrates clear differences in Volta potential that equate to the microstructure of EBM-PBF Ti-6Al-4V as shown in BSE images and EBSD IPFs. In particular, neighboring *α* grains of differing orientations produce differences in Volta potential that are statistically greater than neighboring grains of similar orientation, meaning that misorientation from changes in crystallographic texture has a greater influence on potential than local misorientation from low-angle grain boundaries. In addition, the fraction of relative misorientations and the discrete sets of angles (≈10°, ≈60°, ≈63°, 90°) across *α*–*α* boundaries are consistent with a displacive transformation mechanism,^42,43^ likely due to the fast cooling rates in AM. Although misorientations of *α*–*α* boundaries with differing grain orientation are similar for the orientation categories, the change in Volta potential with respect to misorientation is more severe in *α* laths from {101} and {111} prior-*β* grains as compared to {001} prior-*β* grains. The magnitude of these differences is greatest for *α*–*α* boundaries (113 mV) and *α*–*β* boundaries (152 mV) that originate from {111} prior-*β* grains. The scatter in Volta potential is also larger in *α* laths from {101} and {111} prior-*β* grains, as compared to {001} prior-*β* grains. The increased scatter is likely due to the relatively low strength of these textures and differences in variant selection^42,44^ caused by re-melting of layers in the AM process. It is likely that {111} and {101} prior-*β* grains have a stronger variant selection (specifically the variants^42,45^ that produce misorientation values of ≈60°, ≈63°, and 90°) such that preferred boundary termination of *α* laths creates a larger Volta potential, but differences in populations of interplanar spacings could also be a factor.^46,47^

Interestingly, the {001} *β* grains and *α* laths from {001} prior-*β* grains have the lowest relative difference in Volta potential as well as the lowest maximum Volta potential. In terms of Volta potential magnitude (greatest deviation from the mean), the average maximum value in {111} *β* grains is 118 mV (the greatest of all *β* orientations). The minimum Volta potentials in *α* laths are approximately the same for all orientations (~−65 mV). For context, it is important to note that previous work suggests that 100 mV relative differences are sufficient to drive micro-galvanic corrosion.^27^ Previous studies have correlated dislocation density,^48^ local misorientation/strain localization,^49^ and crystallographic orientation^39^ with substantial differences in Volta potential for cubic crystal structures in wrought steel components and thin films of pure copper. More relevant to the current study is the work completed by Song *et al*.^50,51^ on a wrought Mg alloy (hexagonal close-packed crystal structure) that demonstrates that differences in grain orientation cause changes in the measured work function due to atomic arrangement (preferential attack is more likely on loosely packed planes). The theory provided by Smoluchowski^52^ states that the electron charge distribution, the number of nearest neighbors, and the number of atoms in contact with the surface (dangling bond) influence the work function, and in general, the work function increases as the packing fraction increases. The magnitude of relative Volta potential differences measured in the current work (on an additively manufactured multi-phase alloy) is in line with previous studies and suggests that the surface electronic structure changes as a function of crystal structure and grain orientation, which leads to changes in work function/Volta potential.

Another important finding is that EBM-PBF Ti-6Al-4V parts can contain crystallographic textures that differ from the texture typically assumed for this material, i.e., ⟨001⟩ *β*-fiber aligned with the build direction. This finding is consistent with previous work,^25^ which showed that differences in crystallographic texture lead to measurable changes in macro-scale mechanical properties. On a local scale, an obvious area of concern in AM is the border between contour and hatching microstructures, where significant changes in grain size, phase fraction, and texture have been observed.^53^ In addition, as with some wrought Ti-6Al-4V microstructures, elongated *α* grains tend to form on prior-*β* grain boundaries in EBM-PBF Ti-6Al-4V, causing strain localizations.^54,55^ While maintaining the same features (prior-*β* grain boundaries), it was shown that prior-*β* grain boundaries with differing orientations can exacerbate differences in Volta potential, e.g., if {111} *β* grains are in close proximity to *α* laths and *β* grains that originated from {001} or {101} prior-*β* grains. Locally textured regions have the potential to create anode and cathode couplings on a scale that is much larger than the average grain size. Given the tunability of AM,^56^ recommendations from this work include minimizing {111} prior-*β* grains and macro-textured regions (i.e., groups of {111} prior-*β* grains that neighbor groups of {001} prior-*β* grains). Looking further, these recommendations should apply to other metal AM parts than grains with body-centered-cubic and hexagonal close-packed crystal structures. Future work will focus on face-centered-cubic metals that have been successfully manufactured with additive-based technologies^57^ to probe the differences between subtle changes in texture, sub-grain boundary spacing, and pore structure.

In summary, the microstructure and properties of AM Ti-6Al-4V were analyzed by combining and co-localizing advanced electron microscopy and atomic force microscopy methods. The greatest magnitude of Volta potentials measured in *α* grains and *β* grains originated from {111} prior-*β* grains. Minimal differences were found between {111} and {101} prior-*β* grains, but {001} prior-*β* grains contained a population of Volta potential differences that were significantly lower than the other orientation categories, likely due to the direct relationship between work function and planar density. In a single grain and across *α*–*α* boundaries of differing orientation, those originating from {001} prior-*β* grains exhibited the smallest scatter. Moreover, all *α*–*β* phase boundaries exhibited a relative Volta potential difference equal or greater than *α*–*α* boundaries of dissimilar grain orientation. However, *α*–*β* phase boundaries constitute a small percentage of the total grain boundaries that bisect grains of differing grain orientation. Future studies for AM parts will investigate the surface electronic structure and corrosion potential in other crystal structures and contour–hatch interfaces and harnessing AM technologies to homogenize grain orientations.

## Supplementary Material

Supplementary Material

## Figures and Tables

**FIG. 1.**
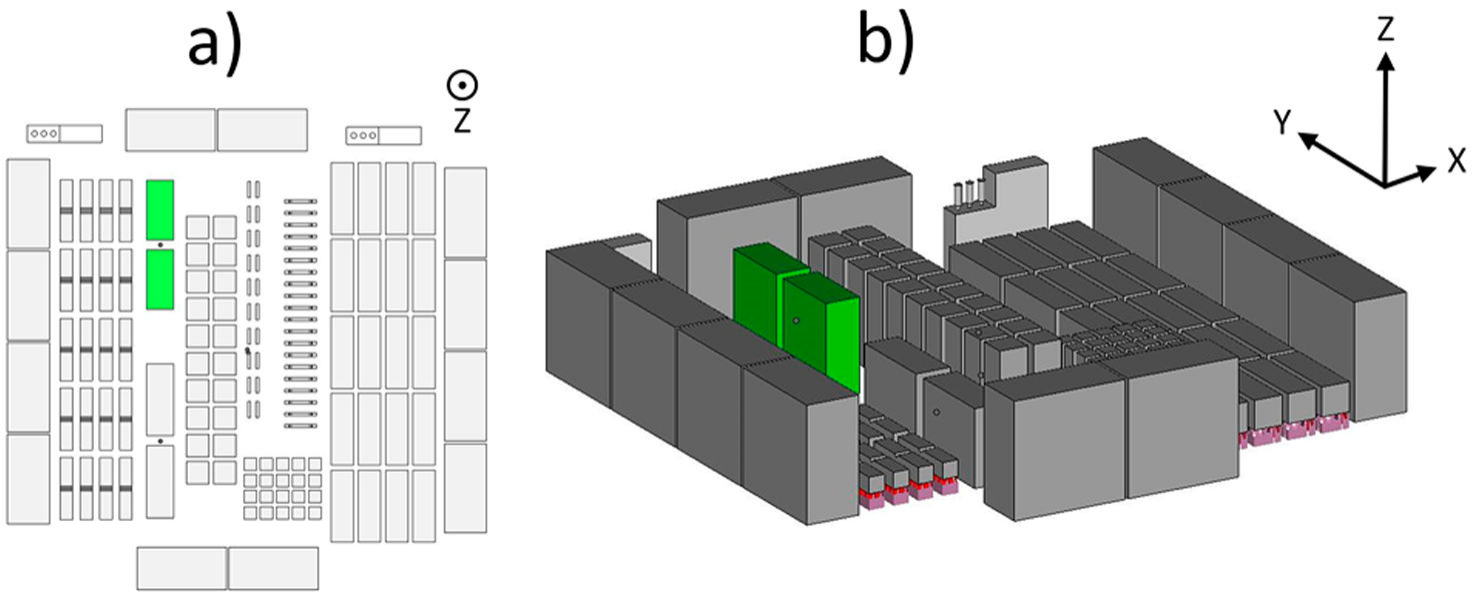
Build layout showing the (a) top view and (b) isometric view to highlight the blocks investigated (green). Z is the build direction.

**FIG. 2.**
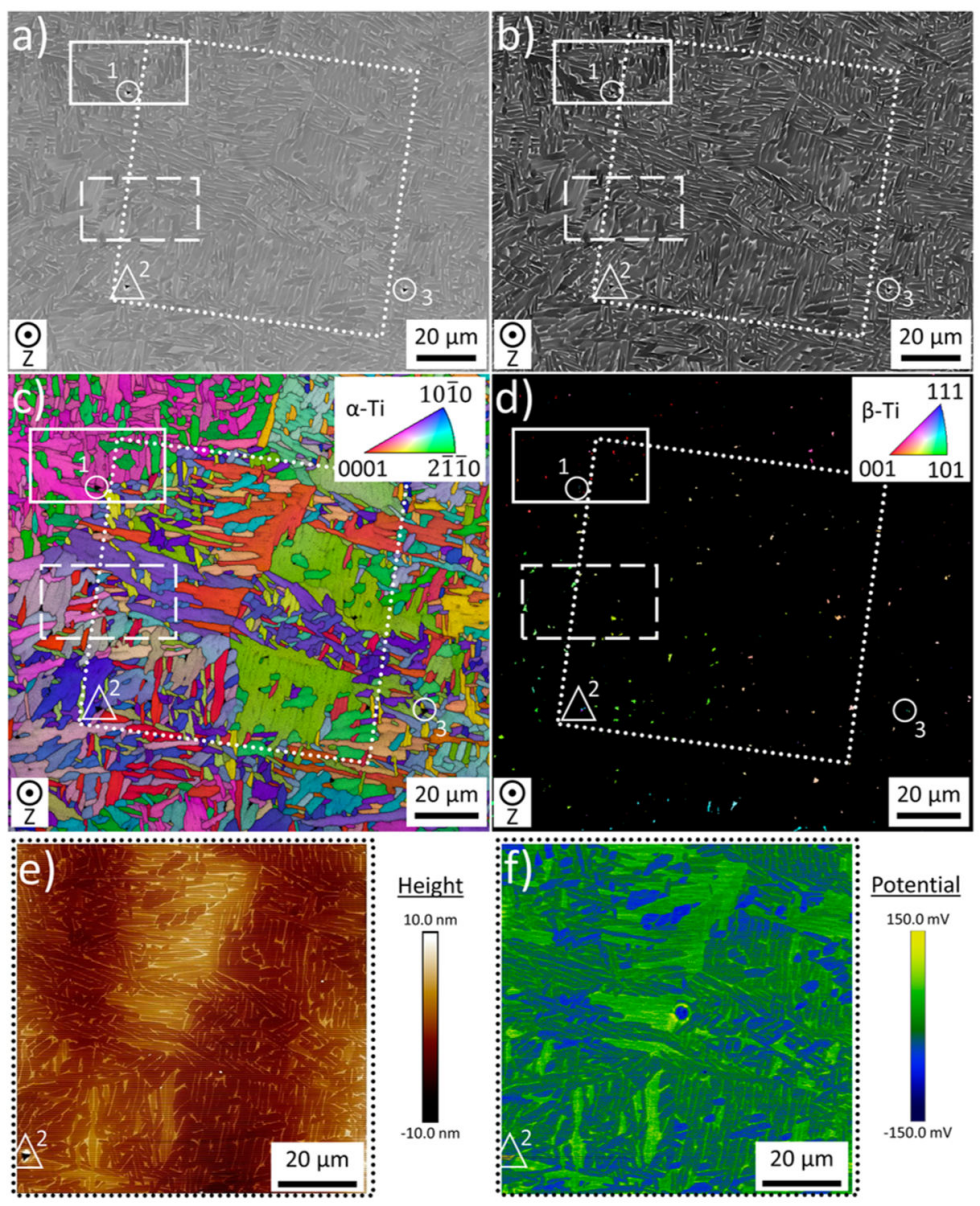
An asymmetric pattern of three fiducial marks (labeled 1–3 and indicated by circles and a triangle) allowed for analysis of the same region of interest using multiple characterization techniques: (a) SE imaging, (b) BSE imaging, and EBSD measurements of (c) *α*-Ti and (d) *β*-Ti. The area indicated by the dotted square in (a)–(d) was subsequently characterized with SKPFM to produce (e) height and (f) Volta potential images. The solid and dashed rectangles in (a)–(d) represent higher resolution SKPFM scans analyzed in greater detail in Figs. 3 and 4.

**FIG. 3.**
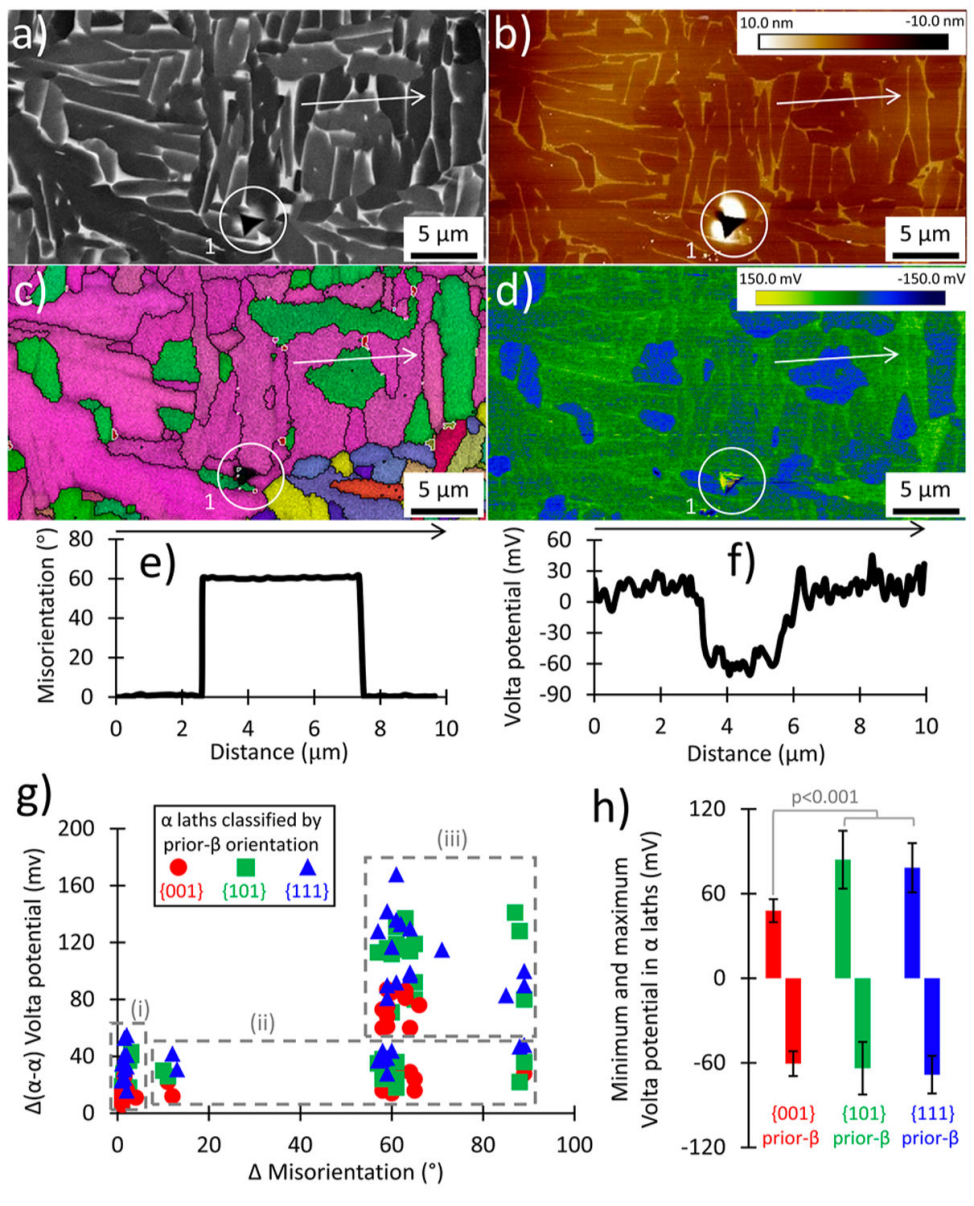
Technique for characterizing *α* laths (area designated by the solid rectangle in Fig. 2) by co-locating: (a) BSE imaging, (b) SKPFM height, (c) EBSD (white lines indicate *α*–*β* phase boundaries, and black lines designate defined grain boundaries), and (d) SKPFM Volta potential. The results from line scans across hypermaps indicated by the white arrows in (a)–(d) are shown for (e) EBSD and (f) SKPFM Volta potential. (g) Summaries of relative differences in Volta potential are shown for three types of measurements: (i) within a single *α* lath, (ii) across *α*–*α* boundaries of similar grain orientation, and (iii) across *α*–*α* boundaries of differing grain orientation. (h) Ranges of Volta potential for different prior-*β* orientations (one standard deviation shown).

**FIG. 4.**
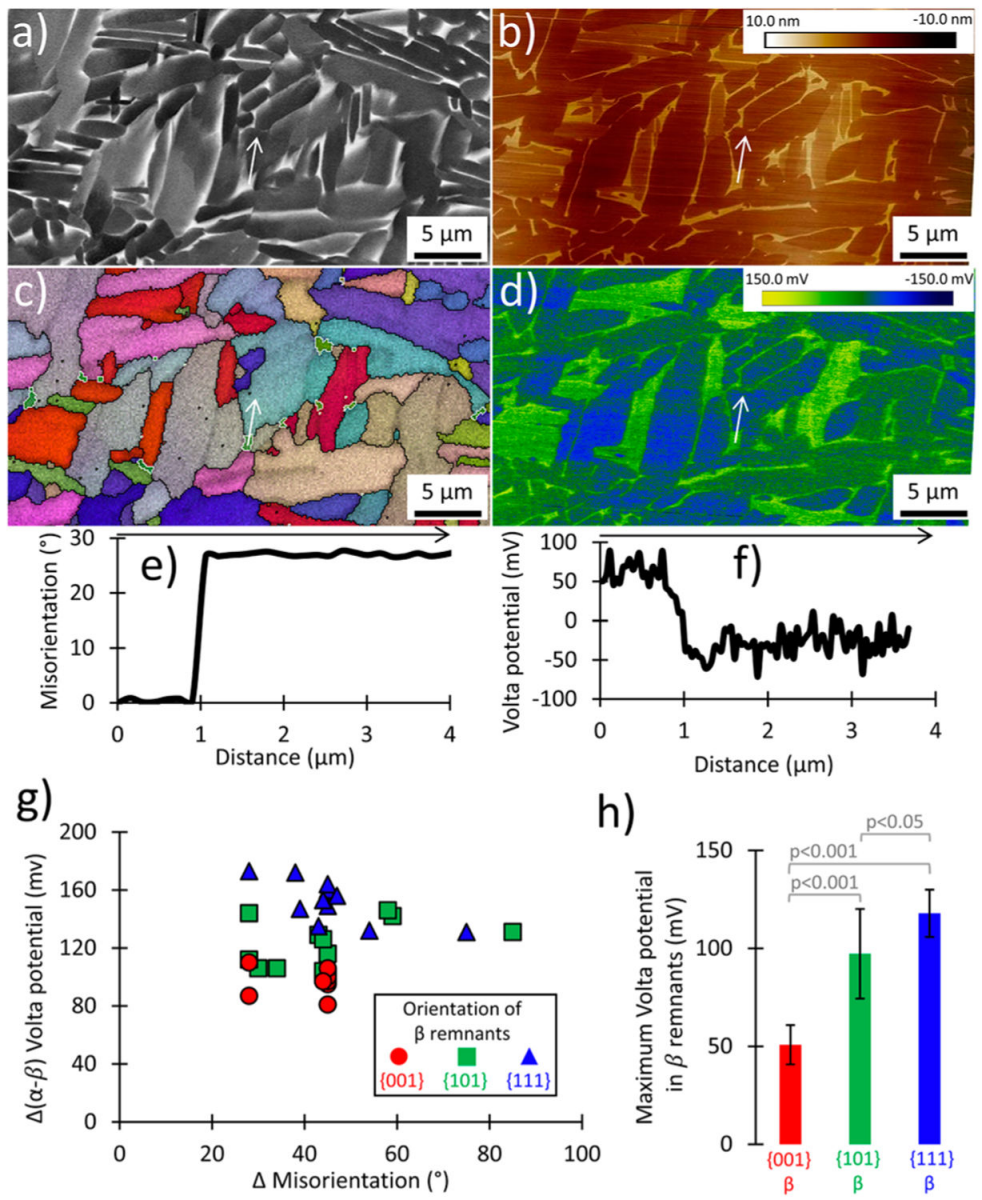
Technique for characterizing *β* grains (area designated by the dashed rectangle in Fig. 2) by co-locating: (a) BSE imaging, (b) SKPFM height, (c) EBSD (white lines indicate *α*–*β* phase boundaries, and black lines designate defined grain boundaries), and (d) SKPFM Volta potential. The results from the region-to-region analysis of the change in (e) misorientation and (f) Volta potential from a *β* rib/grain to the neighboring *α* lath [indicated by the white arrows in (a)–(d)] are shown. Summaries from fields of view in (c) and (d) are provided for all *α*–*β* boundaries, reporting both (g) relative differences in Volta potential and (h) maximum Volta potentials in *β* grains (one standard deviation shown).

**TABLE I.** Chemistry of Ti-6Al-4V parts (mass %) before and after HIP treatments (measurements conform to ASTM B348-13^40^ and ASTM F2924-14^41^).

Material condition	Ti (%)	Al (%)	V (%)	Fe (%)	O (%)	C (%)	N (%)	H (%)
As-built	Balance	5.89	4.4	0.16	0.14	0.01	0.02	0.001
HIP	Balance	5.82	4.3	0.17	0.14	0.01	0.02	0.001

**TABLE II.** Misorientation and Volta potential values from EBSD and SKPFM measurements for single and neighboring *α* grains.

Prior-*β* grain orientation	(i) Δ misorientation, relative difference in Volta potential within a single *α* grain	(ii) Δ misorientation, relative difference in potential across *α* orientation	(iii) Δ misorientation, relative difference in Volta potential across *α* orientation	Minimum Volta potential (mV)	Maximum Volta potential (mV)
{001}	1.6° ± 0.8°, 13 ± 5 mV	58° ± 24°, 23 ± 7 mV	61° ± 3°, 75 ± 10 mV	−61 ± 9	48 ± 8
{101}	1.8° ± 0.8°, 25 ± 11 mV	56° ± 22°, 29 ± 6 mV	66° ± 11°, 112 ± 20 mV	−66 ± 19	84 ± 20
{111}	1.5° ± 0.4°, 34 ± 12 mV	56° ± 23°, 40 ± 6 mV	67° ± 11°, 113 ± 25 mV	−68 ± 13	78 ± 17

**TABLE III.** Misorientation and Volta potential values from EBSD and SKPFM measurements of neighboring *α*–*β* grains.

*β* grain orientation	Δ misorientation, relative difference in Volta potential	Maximum Volta potential
{001}	41° ± 7°, 97 ± 9 mV	51 ± 10
{101}	45° ± 16°, 121 ± 17 mV	97 ± 23
{111}	46° ± 12°, 152 ± 15 mV	118 ± 12

## Data Availability

The data that support the findings of this study are available from the corresponding author upon reasonable request.
