# Supplementary material for: Impact of grain orientation and phase on Volta potential differences in an additively manufactured titanium alloy
Source: AIP Adv. Author manuscript; Available in PMC 2021 Jul 10. (PMC8272250; doi:10.1063/5.0038114)
Supplement: Supplementary Material [file NIHMS1714427-supplement-Supplementary_Material.docx]

Supplementary Material

Impact of grain orientation and phase on Volta potential differences in an additively-manufactured titanium alloy

Jake T. Benzing,^1^* Olivia O. Maryon,^2^ Nik Hrabe,^1^ Paul H. Davis,^2^ Michael F. Hurley,^2^ and Frank W. DelRio^1^

^1^ Material Measurement Laboratory, National Institute of Standards and Technology, Boulder, CO 80305, USA

^2^ Micron School of Materials Science and Engineering, Boise State University, Boise, ID 83725, USA

*corresponding author. Please contact at [jake.benzing@nist.gov](mailto:jake.benzing@nist.gov)

# SKPFM Methods

Scanning Kelvin Probe Force Microscopy (SKPFM) imaging experiments were performed in an inert argon atmosphere containing < 0.1 ppm H_2_O and O_2_ using a Bruker Dimension Icon AFM (Bruker Nano, Santa Barbara, USA) housed in an MBraun glovebox (MBraun, Stratham, USA). SKPFM generates a <100 nm resolution map of the tip-sample Volta potential difference as a nanoscale conductive probe rasters tens to hundreds of nanometers above the sample surface. In our experiments, a PFQNE-AL probe with a silicon nitride cantilever and an uncoated doped silicon tip (Bruker PFQNE-AL, *k* = 0.8 N/m, *f_0_* = 300 kHz) was used for all topography and Volta potential measurements. SKPFM was performed using a dual-pass lift off mode. The first pass consisted of PeakForce Tapping along the surface to acquire a topography map. Upon completing the first pass, the probe was lifted off the surface to a user-defined height (25 nm in these experiments). During the second pass to measure the voltage potential difference between the sample and the probe, the topography was then retraced at this 25 nm lift height above the surface while a variable DC bias was applied to the sample to null the tip-sample potential difference at each data point.

An SKPFM standard provided by Bruker was imaged before and after imaging the Ti-6Al-4V sample. The standard consists of an n-doped Si wafer covered with a 50 nm thick Au layer and patterned with an array of 50 nm thick rectangular Al islands surrounded by “moats” of bare Si. Measurement of the tip-Au Volta potential difference allows for quantitative analysis of SKPFM Volta potentials and confirmed the tip’s work function remained constant throughout the course of experiments.

The Ti-6Al-4V samples were brought into the glovebox and imaged as provided/received. To co-localize the EBSD and SEM imaging conducted prior to the SKPFM, a Sharpie mark and intentional scratch in the Bakelite mount were placed at the designated top section of the sample. From the center of the mark, we navigated down to a set of three fiducial marks, ~750 µm away from the edge of the Ti-6Al-4V. The fiducial marks were placed in a triangular pattern, with length 70 µm and width 100 µm. The left corner mark served as the focus/origin. From there, SKPFM images 40 µm × 40 µm and 90 µm × 90 µm in size were obtained. Throughout, the scan parameters were:

| **Parameter** | **Value** |
| --- | --- |
| Feedback Gain | 4 |
| Peak Force Setpoint | 2.2 nN |
| Lift Height | 25 nm |
| Integral Gain | 3 |
| Potential Gain | 5 |
| Scan Rate | 0.200 Hz |
| Samples/Line (40 µm) | 1024 |
| Samples/Line (90µm) | 2048 |
| Peak Force Tapping Amplitude | 80 nm |

As mentioned above, the Volta potential difference between the conductive probe and the surface is quantified by applying a variable DC bias to null the tip-sample electric force gradient arising from the voltage potential difference (VPD) between the probe and surface. Nulling of the electric force gradient is confirmed by varying the applied DC bias to minimize the probe oscillations at the sum and difference frequencies due to simultaneous mechanical oscillation of the probe at its natural resonance frequency and electrical biasing at 2 kHz (V_AC_ = 5 V) while suspended near (25 nm above) the sample surface. Through using the SKPFM setup, VPD maps were acquired. These VPD maps were compared to the EBSD and SEM images taken to understand where and why galvanic corrosion is likely to occur on the sample.

# SKPFM Data Processing

NanoScope Analysis Version 1.9 was used to process all AFM images. For the SKPFM images, a stop band was placed over the fiducial marks as well as any obvious pieces of dust/debris. Large area (90 µm × 90 µm) and medium area (40 µm × 40 µm) images of the AM Ti-6Al-4V sample were processed with a combination of plane fit and flatten filters. Typically, first and second order polynomial filters are used on height sensor maps to account for the effects of sample tip, tilt, and curvature; any bow that may arise due to the piezo tube scanner; and/or pressure differences from periodic refills of the gaseous environment housed in the glove box that can cause apparent height differences along the slow scan axis direction. Additionally, line-to-line offsets sometimes arise in a closed loop AFM sensor due to the SKPFM tip picking up or dropping debris (or micro-fractures in the tip). In the case of charged debris on the tip, line-to-line offsets can occur in the potential channel as well.

Beyond line-to-line offsets, some curvature in the potential channel maps was also observed, possibly due to charging of the probe or sample during slow SKPFM scans (which could take hours due to the dual pass mode and high lateral resolution steps). However, Volta potentials measured during SKPFM lift mode were relatively insensitive to the SKPFM feedback gain. The magnitude of spread in Volta potential values are of interest for the current work, since these are what give rise to Volta potential differences between adjacent grains. A 1^st^ order flatten filter and 2^nd^ order plane fit filter were applied to the data and the minima and maxima were reported with respect to the mean. When comparing filter combinations, no significant differences in surface roughness (*i.e.*, surface potential standard deviation within a grain) were measured.

**SKPFM Limitations**

The limitations of the analysis methods employed in this work, which are primarily related to the resolution limits of each characterization technique, must be discussed. Specifically, differences in Volta potential measured by SKPFM tend to underestimate the true difference when comparing adjacent grains and phases due to spatial averaging for measurements on length scales near the resolution limit. To minimize this effect of spatial averaging across or near a boundary, the relative Volta potential differences between α-α grain boundaries and α-β phase boundaries were computed using values that were obtained at least 1 µm from a given boundary. In particular, characterizing α-β phase boundaries proved to be the greatest challenge due to the size (sub-μm) of remaining β remnants. Although EBSD measurements with a 150 nm step size still provided confident identification of many β remnants, care was taken to only cross one sharp phase boundary for a given line scan.

One other consideration is the possible presence of topographical artifacts in Volta potential measurements. Although the vibratory polish resulted in an extremely smooth mirror-like finish, with only ~7 nm differences in topography between adjacent α and β phases, an SKPFM lift height investigation was performed employing a range of lift heights from 15 nm to 50 nm. Lift heights ≥25 nm yielded similar Volta potentials, whereas a 15 nm lift height showed evidence of some phase jumps due to the tip contacting the surface during the lift mode pass based on the chosen SKPFM oscillation amplitude. Ultimately, a 25 nm lift height provided the best balance between resolution and signal-to-noise without resulting in topographical artifacts in the measured Volta potentials. This extremely low lift height was only possible because of the sample’s minimal surface roughness (R_q_ of approximately 3 nm) and conducting the measurements in a glovebox, thereby preventing formation of the layer of surface water present under ambient conditions that can cause tip-sample adhesion issues and limit the minimum usable lift height.
